# Plant-Derived Antiviral Compounds as Potential Entry Inhibitors against Spike Protein of SARS-CoV-2 Wild-Type and Delta Variant: An Integrative in SilicoApproach

**DOI:** 10.3390/molecules27061773

**Published:** 2022-03-08

**Authors:** Jenifer Mallavarpu Ambrose, Malathi Kullappan, Shankargouda Patil, Khalid J. Alzahrani, Hamsa Jameel Banjer, Fadi S. I. Qashqari, A. Thirumal Raj, Shilpa Bhandi, Vishnu Priya Veeraraghavan, Selvaraj Jayaraman, Durairaj Sekar, Alok Agarwal, Korla Swapnavahini, Surapaneni Krishna Mohan

**Affiliations:** 1 Department of Research, Panimalar Medical College Hospital & Research Institute, Chennai 600123, India; jenifer.pmchri@gmail.com (J.M.A.); malak.hari@gmail.com (M.K.); 2 Department of Maxillofacial Surgery and Diagnostic Sciences, Division of Oral Pathology, College of Dentistry, Jazan University, Jazan 45412, Saudi Arabia; dr.ravipatil@gmail.com; 3 Department of Clinical Laboratories Sciences, College of Applied Medical Sciences, Taif University, P.O. Box 11099, Taif 21944, Saudi Arabia; ak.jamaan@tu.edu.sa (K.J.A.); h.banjer@tu.edu.sa (H.J.B.); 4 Department of Microbiology, College of Medicine, Umm Al-Qura University, Makkah 24381, Saudi Arabia; fsqashqari@uqu.edu.sa; 5 Department of Oral Pathology and Microbiology, Sri Venkateswara Dental College and Hospital, Chennai 600130, India; thirumalraj666@gmail.com; 6 Department of Restorative Dental Science, Division of Operative Dentistry, College of Dentistry, Jazan University, Jazan 45142, Saudi Arabia; shilpa.bhandi@gmail.com; 7 Centre of Molecular Medicine and Diagnostics (COMManD), Department of Biochemistry, Saveetha Dental College, Saveetha Institute of Medical and Technical Sciences (SIMATS), Saveetha University, Chennai 600077, India; drvishnupriyav@gmail.com; 8 Centre for Cellular and Molecular Research, Saveetha Dental College & Hospitals, Saveetha Institute of Medical & Technical Sciences (SIMATS), Saveetha University, Chennai 600077, India; duraimku@gmail.com; 9 Department of Chemistry, Chinmaya Degree College, BHEL Haridwar 249403, India; agarwalalok547@gmail.com; 10 Department of Biotechnology, Dr B.R. Ambedkar University, Etcherla, Srikakulam 532410, India; swapnavahini@gmail.com; 11 Departments of Biochemistry, Molecular Virology, Research, and Clinical Skills & Simulation, Panimalar Medical College Hospital & Research Institute, Chennai 600123, India

**Keywords:** molecular modeling, SARS-CoV-2, delta variant, COVID-19, spike glycoprotein, antiviral agents, phytochemical compounds, virtual screening, molecular dynamics and simulations

## Abstract

The wild-type SARS-CoV-2 has continuously evolved into several variants with increased transmissibility and virulence. The Delta variant which was initially identified in India created a devastating impact throughout the country during the second wave. While the efficacy of the existing vaccines against the latest SARS-CoV-2 variants remains unclear, extensive research is being carried out to develop potential antiviral drugs through approaches like in silico screening and drug-repurposing. This study aimed to conduct the docking-based virtual screening of 50 potential phytochemical compounds against a Spike glycoprotein of the wild-type and the Delta SARS-CoV-2 variant. Subsequently, molecular docking was performed for the five best compounds, such as Lupeol, Betulin, Hypericin, Corilagin, and Geraniin, along with synthetic controls. From the results obtained, it was evident that Lupeol exhibited a remarkable binding affinity towards the wild-type Spike protein (−8.54 kcal/mol), while Betulin showed significant binding interactions with the mutated Spike protein (−8.83 kcal/mol), respectively. The binding energy values of the selected plant compounds were slightly higher than that of the controls. Key hydrogen bonding and hydrophobic interactions of the resulting complexes were visualized, which explained their greater binding affinity against the target proteins—the Delta S protein of SARS-CoV-2, in particular. The lower RMSD, the RMSF values of the complexes and the ligands, Rg, H-bonds, and the binding free energies of the complexes together revealed the stability of the complexes and significant binding affinities of the ligands towards the target proteins. Our study suggests that Lupeol and Betulin could be considered as potential ligands for SARS-CoV-2 spike antagonists. Further experimental validations might provide new insights for the possible antiviral therapeutic interventions of the identified lead compounds and their analogs against COVID-19 infection.

## 1. Introduction

The COVID-19 pandemic situation has urged the healthcare experts and biomedical scientists to conduct several studies for understanding the mechanism of infection, disease progression, prevention, and therapeutics, throughout the world. Despite the progress made, the complexity of the disease transmission and presentation has increased, which leads to higher mortality rates. The genome of the SARS-CoV-2 virus, like other RNA viruses, undergoes a rapid evolution, resulting in the emergence of multiple variants, as reported in different regions across the world [1]. Some of the mutations in their genome are speculated to be deleterious, which could affect the functional attributes of the associated genes and proteins. Thus, such deleterious mutations may modify the transmissibility, disease severity, or interactions of the pathogen with the host immune system. Those novel strains have not only manifested varying symptoms but are also suspected to impact the effectiveness of the prophylactic vaccines [2,3]. The Delta variant, which was first identified in India, has been linked to the resurgence of COVID-19 cases and millions of deaths during the second wave in India and a few other countries between March 2021 and May 2021 [4,5]. When an antiviral inhibitory molecule is bound to its target, it is believed to have a direct (virucidal) effect on the virus itself [6]. This serves as the rationale behind the selection of spike RBD as the principal target macromolecule in this study.

**Figure 1:**
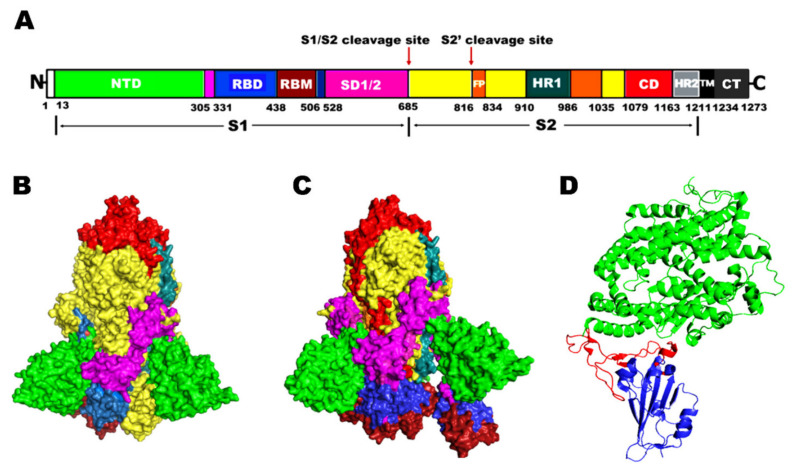
(**A**) Schematic representation of the domains arranged in the structure of the SARS-CoV-2 Spike protein. Different domains that constitute the protein are color-coded. NTD, N-terminal domain; RBD, Receptor binding domain; RBM, Receptor binding motif; SD1/2, Subdomain 1 and 2; S1/S2, protease cleavage sites S1 and S2; FP, Fusion peptide; HR1, Heptad repeat 1; CH, Central helix; CD, Connector domain; HR2, Heptad repeat 2; TM, Transmembrane domain; CT, Cytoplasmic tail. The protease cleavage (polybasic) sites located at the interface of S1 and S2 subunits are indicated by arrows, wherein the viral and host cell membrane fusion takes place. (**B**) Cryo-EM structure of the SARS-CoV-2 S glycoprotein homotrimer (PDB: 7DF3) in closed conformation (**C**) Cryo-EM structure of the SARS-CoV-2 S glycoprotein homotrimer (PDB: 7DK3) in open conformation, and (**D**) Secondary structure of S RBD bound to human ACE2 (PDB: 6M0J). Core RBD is shown in blue, RBM in red, and ACE2 in green.

The spike glycoprotein in the proteome of the SARS-CoV-2 virus establishes trimers on its surface, which are responsible for the recognition of the ACE2 receptor, entry, and subsequent fusion into the human host [7]. While establishing the infection, the metastable pre-fusion state of the S protein is mediated by the non-covalent interactions that link subunits 1 and 2 (S1 and S2) (Figure 1). The S1 subunit, which is 672 amino acids long, comprises four domains—an N-terminal domain (NTD), a receptor-binding domain (RBD) and the subdomains 1 and 2 (SD1 and SD2) [7]. RBD is an appealing target macromolecule in the SARS-CoV-2 drug discovery process, as it serves as the intermediary factor in the virus–host cell interactions. Particularly, the interaction between the receptor-binding motif (RBM) of RBD with the angiotensin-converting enzyme 2 (ACE2) receptor drives the viral infection process by inducing the transition of the Spike protein from a metastable pre-fusion state to a more stable post-fusion state. This change is crucial for the membrane fusion that has been observed between the host cell and the pathogen. On the other hand, the 588 amino-acid-long S2 subunit consists of a fusion peptide (FP) at its N-terminal and two heptad repeats (HR1 and HR2) that facilitate the S2 subunit to associate with the host membrane. Apart from these, the S2 domain also contains a transmembrane domain (TM) and a cytoplasmic tail (CT), which are involved in the attachment of the S protein to the membrane of the virus [7,8,9]. Blocking the viruses like SARS-CoV-2 influence them to continuously evolve as genetic mutations occur during their genomic replication process.

The SARS-CoV-2 spike protein is a class I viral protein that plays a pivotal role in the viral entry mechanism by facilitating the fusion of viral and host cell membranes [10,11]. The variants reported, including Delta, Gamma, Beta, and Alpha, possess mutations in the receptor-binding domain of the spike protein, which increased the binding affinity of the spike glycoprotein to the ACE2 host receptor [12]. T19R, T95I, G142D, E156-, F157-, R158G, A222V, W258L, K417N, L452R, T478K, D614G, P681R, and D950N were the mutation hotspots reported in the SARS-CoV-2 Delta variant [13]. Hence, this study attempted to employ virtual screening (VS) and molecular docking techniques for identifying promising plant-based drug candidates that could inhibit the spike protein of the SARS-CoV-2 wild-type as well as the Delta variant.

## 2. Results

**Figure 2:**
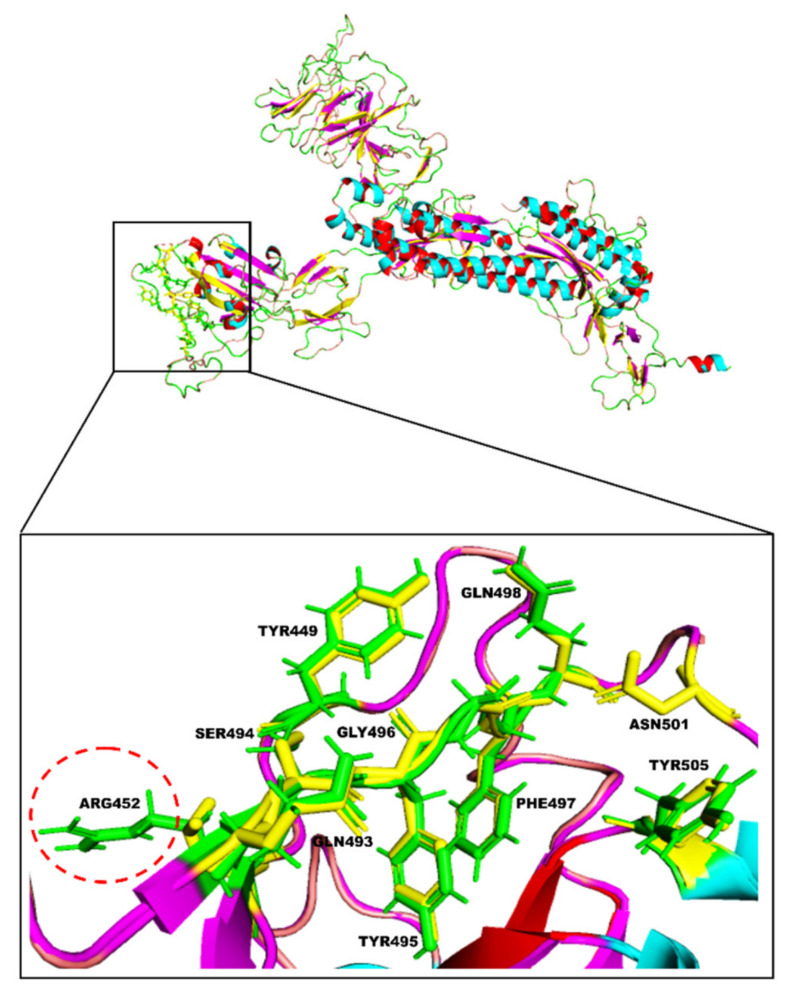
Superimposed 3D structures representing the spike RBD region of wild-type (yellow) and Delta (green) SARS-CoV-2 strains with their active site residues highlighted. L452R (substitution in Delta) and N501Y (substitution present only in wild-type) are the two mutations reported in the RBD region of the two variants.

**Figure 3:**
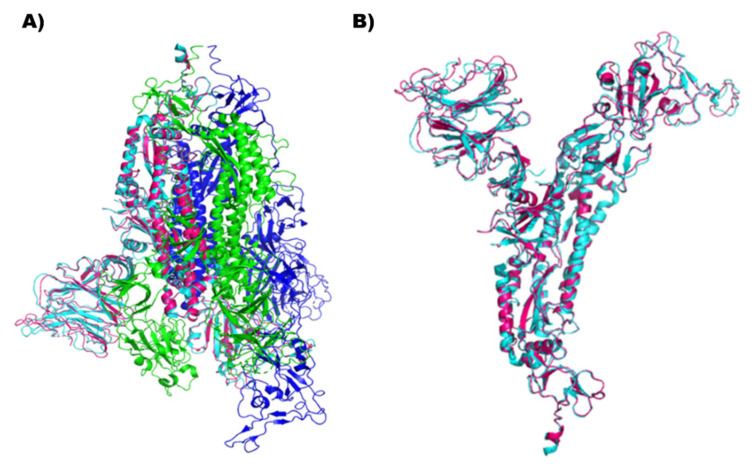
Superimposed snapshot of the modelled and the crystal structure (PDB: 7W92) of the Delta S RBD. (**A**) S RBD crystal structure of 7W92, a homotrimer, with the chains A, B, and C highlighted in blue, cyan, and green, respectively. Here, the modeled Delta S RBD aligned with the B chain of 7W92 after superimposition is highlighted in cyan and pink. (**B**) Enlarged view of the superimposed structures of modeled Delta S RBD on 7W92, chain B crystal structure, highlighted in cyan and pink, respectively.

**Figure 4:**
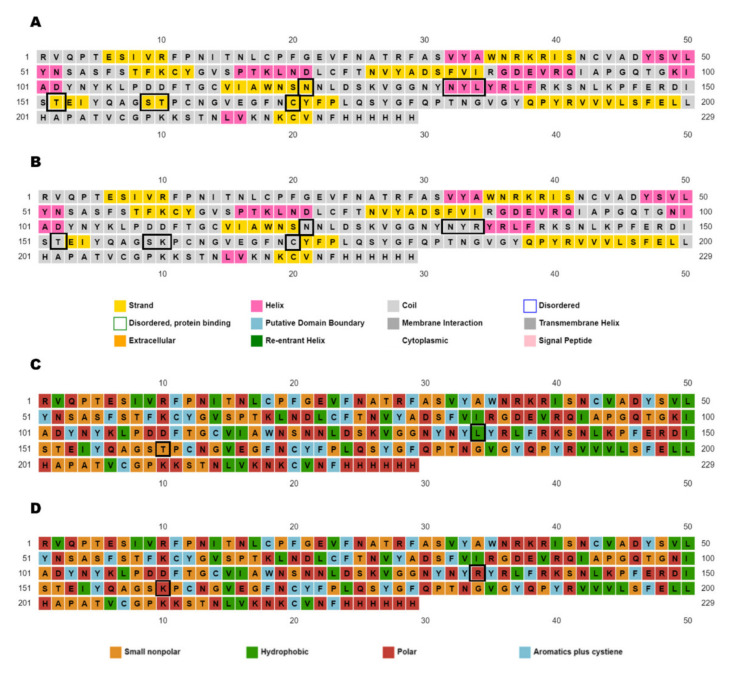
Secondary structure prediction output given by PSIPRED for S RBD of (**A**) wild-type and (**B**) Delta forms of SARS-CoV-2. (**C**,**D**) represent the changes in the formation of secondary structural elements between the two protein sequences due to amino acid substitutions, and deletions are highlighted in the black boxes.

In the present computational study, the inhibitory effect of natural compounds previously identified in Indian medicinal plants such as Decalepis hamiltonii, Acacia modesta, Hypericum perforatum, Phylanthus amarus, and Phyllanthus emblica was investigated against the SARS-CoV-2 Spike glycoprotein using a traditional structure-based docking approach. The overall amino acid sequence similarity between the two forms of S RBD was 97.0% (data not shown). The RMSD value computed between the atoms of the superimposed macromolecular structures of wild-type and mutant forms was 0.227 Å, which indicates their structural similarity and compatibility for further computational analysis. The similarities and differences between the amino acid residues constituting the active site were visualized in their superimposed 3D structures; particularly, the loop region spanning the receptor-binding motif (RBM) of their RBDs was examined between the positions 449 and 505, which showed a mismatch at 452 (Figure 2). Likewise, the modeled 3D structure of the Delta S RBD was superimposed with the recently resolved crystal structure 7W92 (Figure 3). The RMSD value obtained for the superimposed models was 0.858Å, which confirmed the reliability of the model obtained for the study. From the secondary structure prediction results, subtle changes were noticed in the formation of secondary structural elements between their S RBD structures due to amino acid substitutions observed in the Delta variant. Our results revealed that the amino acid substitutions (L452R and T478K) modified alpha-helix and beta-strand structures to the coil, as highlighted in Figure 4A,B. In addition, the two amino acid substitutions have influenced four other neighboring amino acids, whose secondary structures have changed from beta-strand to coil. The substituted amino acids conferred to specific secondary structures in the two S RBD proteins. As depicted in Figure 4C,D, the substitutions in the S RBD region of the Delta variant were found to have gained two polar amino acids (L at 452 and T at 478), which were previously known to be of hydrophobic and small non-polar types.

### 2.1. Efficient Antiviral Inhibitory Compounds Screened against SARS-CoV-2 RBD

We virtually screened a library of fifty phytochemical compounds against the spike glycoprotein of SARS-CoV-2 to discover a possible therapy for the cure of the COVID-19 infectious disease. AutoDock Vina incorporated in the PyRx tool generated nine different binding conformations for each ligand, which were subsequently ranked by their binding energy. While the screening against the wild-type Spike protein showed the binding energy of the top 20 compounds in the range of −9.4 to −7.8 kcal/mol, the drug screening against the Spike protein of the Delta variant displayed the binding energy of the top twenty compounds between −7.5 to −4.9 kcal/mol. The results of the VS process performed against the wild-type and Delta Spike proteins are cataloged in Supplementary Tables S1 and S2, respectively. It was observed that Lupeol, Betulin, Hypericin, Corilagin, and Geraniin topped in the computational drug screening lists of both Spike variants. Nevertheless, the order of the compounds in the complex with the Spike RBD of the two strains slightly varied based on their binding energy values.

### 2.2. Binding Interactions of Potential Antiviral Plant Compounds in the Active Site of the Wild-Type Spike Protein

**Figure 5:**
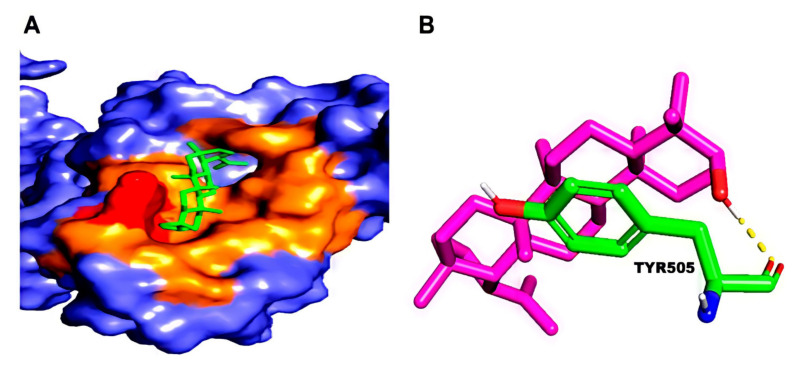
(**A**) Binding pose of the top-ranked phytochemical compound, Lupeol, on the surface of the wild-type Spike RBD. (**B**) 3D view of the molecular interaction between Lupeol and the residues inside the active site of wild-type SARS-CoV-2 S RBD. The hydrogen bond in the protein-ligand complex is represented by a yellow dashed line.

**Figure 6:**
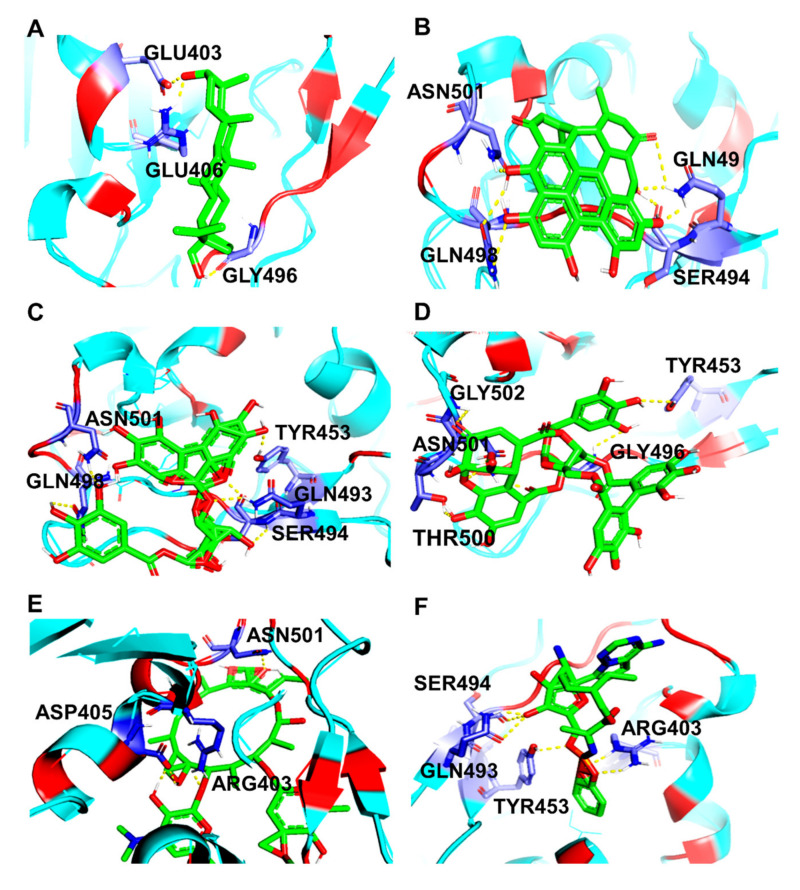
3D diagram of S RBD of wild-type SARS-CoV-2 in complex with (**A**) Betulin, (**B**) Hypericin, (**C**) Corilagin, (**D**) Geraniin, (**E**) Remdesivir, and (**F**) Azithromycin. The interacting amino acid residues and the ligand molecules in the protein-ligand complexes are illustrated as blue and green sticks, respectively. The yellow dashed line represents the hydrogen bond in the protein-ligand complex.

Docking simulations demonstrated that Lupeol strongly binds within the binding pocket of the protein with a binding energy value of –8.54 (Figure 5). The oxygen atom in the hydroxyl group attached to the benzene ring of Lupeol interacted with the aromatic amino acid, TYR505 (H-bond), in the RBD of the wild-type S protein. However, all the ligands interacted bound at the sites ARG403, ASP405, TYR449, TYR453, GLN493, SER494, GLN498, and ASN501 through hydrogen bond interactions (Figure 6). Additionally, residues such as GLU406, LYS417, TYR453, LEU455, TYR495, GLY496, ASN501, and TYR505 were observed to be participating in the hydrophobic interactions. Table 1 presents the molecular interaction data of the wild-type S RBD with the selected ligands in terms of binding energy and the hydrogen bonds formed. The interactions exhibited by Betulin (–8.29 kcal/mol), Hypericin (–8.13 kcal/mol), and Corilagin (–7.21 kcal/mol) were slightly stronger with lower binding energies than that of the synthetic controls like Azithromycin (–7.17 kcal/mol) and Remdesivir (–6.69 kcal/mol), whose binding energies were higher than that of the phytochemical compounds (Table 1).

### 2.3. Binding Interactions of Potential Antiviral Plant Compounds in the Active Site of the Delta Spike Protein

**Figure 7:**
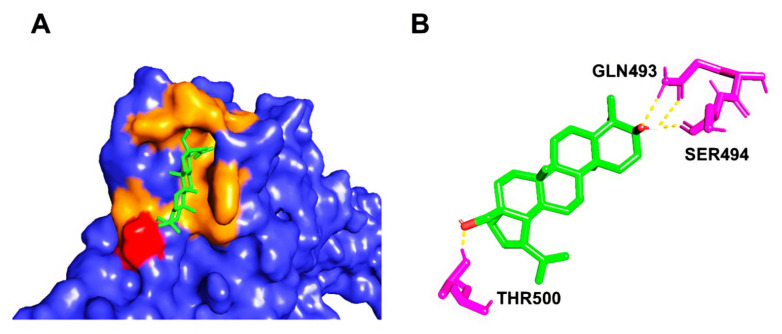
(**A**) Binding pose of the top-ranked phytochemical compound, Betulin, on the surface of Delta Spike RBD. (**B**) 3D view of the molecular interaction between Betulin and the residues inside the active site of wild-type SARS-CoV-2 S RBD. The yellow dashed line represents the hydrogen bond in the protein-ligand complex.

**Figure 8:**
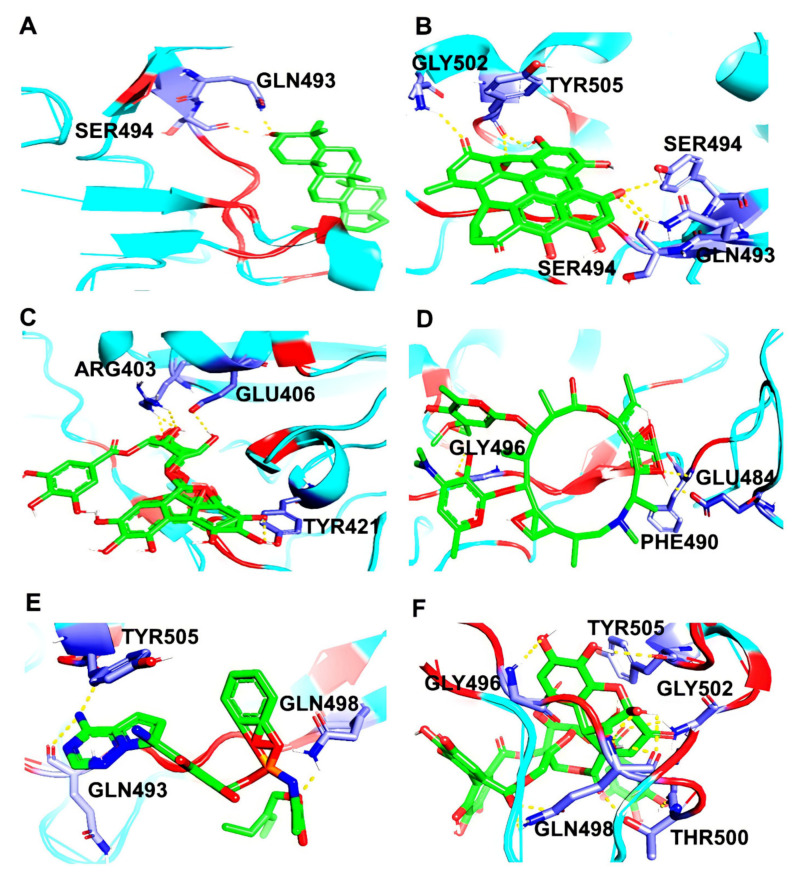
3D diagram of SARS-CoV-2 Delta S RBD in complex with (**A**) Lupeol, (**B**) Hypericin, (**C**) Corilagin, (**D**) Geraniin, (**E**) Remdesivir, and (**F**) Azithromycin. The interacting amino acid residues and the ligand molecules in the protein-ligand complexes are illustrated as blue and green sticks, respectively. The yellow dotted lines represent the hydrogen bonds formed between the protein and the ligand.

Post docking, the best-docked poses of the selected natural compounds evaluated for Delta Spike RBD displayed results similar to that of the wild-type S protein. In general, all of the plant-derived compounds in complex with the mutant S protein yielded the lowest binding energy values compared to that of the wild-type S protein. Among the five compounds, Betulin displayed the highest binding affinity, with a binding energy value of –8.83 kcal/mol (Figure 7). Key molecular interactions of the possible plant-based candidate molecules with the Delta S protein were analyzed. It was evident that the ligand interacted with three residues in the active site of the Delta S protein at THR500, GLN493, and SER494. In the case of wild-type S protein docking interactions, Lupeol was ranked at the top, with the highest binding affinity, whereas the Delta S RBD docking interactions showed that Lupeol was ranked second. It formed hydrogen bonds at GLN493 and SER494 with a binding energy value of −8.68 kcal/mol. The best-docked pose for each ligand with the target protein is illustrated in Figure 8. Similarly, the hydrogen and hydrophobic interactions that were involved in the complex formation were recorded (Table 2). While Hypericin and Corilagin showed a moderate binding affinity, Azithromycin and Remdesivir exhibited less binding affinity to the Delta S RBD, with a relatively lesser number of hydrogen bonding interactions. In both cases, Geraniin showed weak interactions with the lowest binding affinity (–5.91 kcal/mol).

### 2.4. Validation of Docking by MD Simulation

The best complexes obtained were further validated by molecular dynamics and simulation (for a duration of 100 ns) to understand their structural stability in a real-time environment. The trajectories of RMSD, RMSF, Rg, and H-bonds, were graphically analyzed to determine the stability of the protein-ligand wild-type and Delta SARS-CoV-2. Based on their stability, compactness, and ligand contacts examined, the wild-type S RBD-Lupeol and Delta S RBD-Betulin complexes were found to be more stable throughout the simulation process and were explored further in detail.

### 2.5. Wild-Type Spike RBD-Lupeol Complex

**Figure 9:**
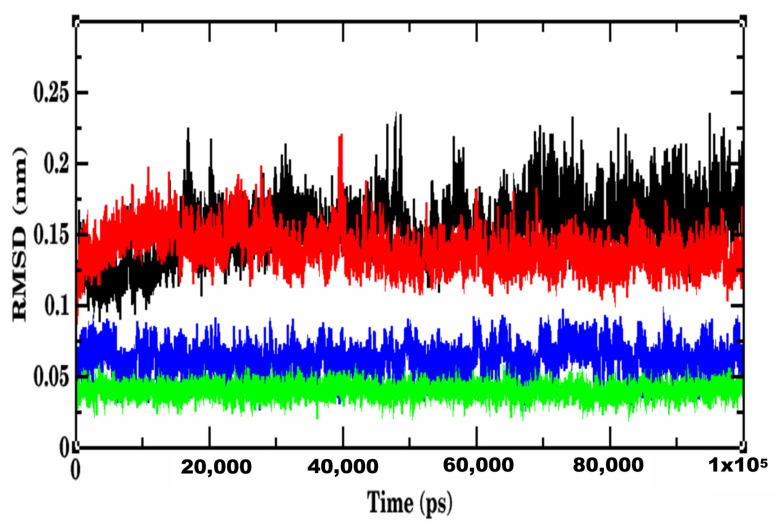
RMSD plot of 100, 000 ps MD simulation showing the trajectory snapshots at different time points. RMSD of protein backbone: the wild-type S RBD-Lupeol complex (black) showed a moderate deviation in the backbone of the protein associated with the complex, when compared to that of the Delta S RBD-Betulin complex (red), RMSD of ligands: the Lupeol that is in complex with Wild-type S RBD (black) showed a moderate deviation, when compared to that of the Lupeol (blue) and Betulin bound to Delta S RBD (green).

**Figure 10:**
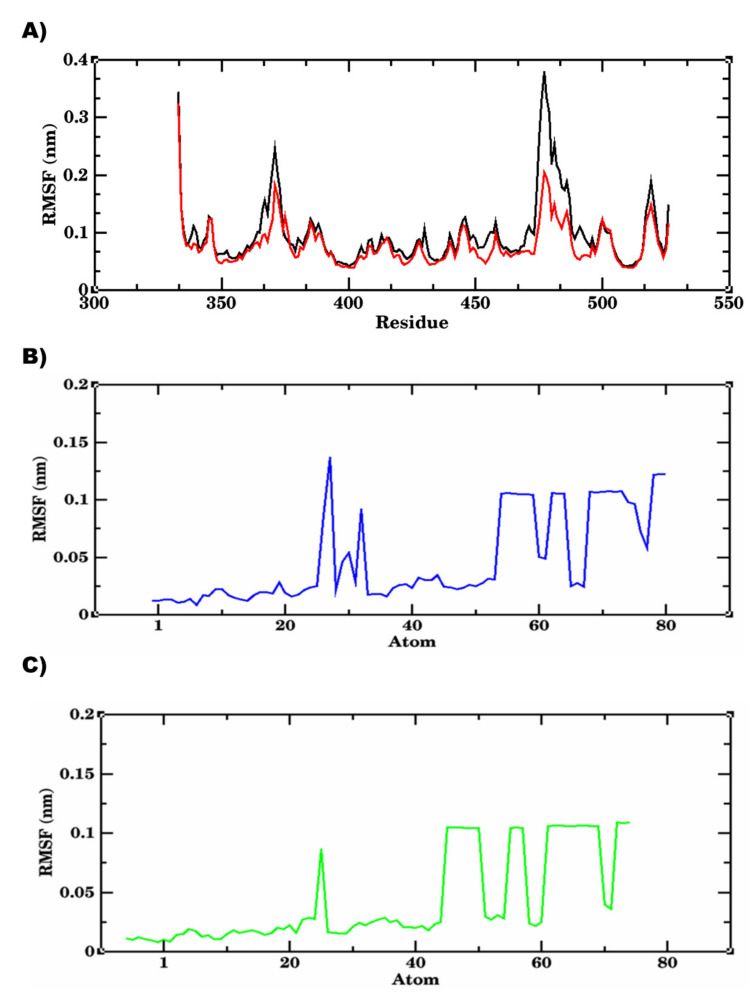
Protein backbone RMSF plot of 100, 000 ps MD simulation depicting the residue-wise fluctuations in the protein-ligand complexes against the RMSF values. (**A**) the residues of wild-type S RBD in complex with Lupeol (black) showed comparatively moderate fluctuations when compared to those of Delta S RBD (red) (**B**) RMSF of Lupeol (blue), showing minimum fluctuations and (**C**) the RMSF of Betulin (green), showing the lowest fluctuations, during the 100,000 ps simulation.

**Figure 11:**
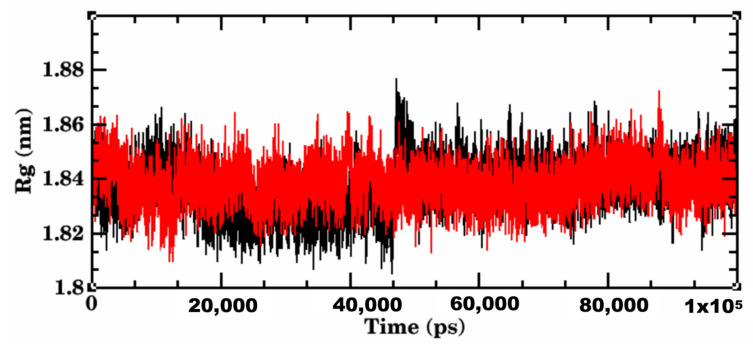
Radius of gyration plot of 100, 000 ps MD simulation illustrating the compactness of the protein-ligand complexes of wild-type-Lupeol (black) and Delta-Betulin (red). No changes were observed in the compactness of both the complexes.

The simulation system of wild-type S RBD-Lupeol consisted of 32 atoms with 13,413 water molecules. When it was neutralized further, 2 Cl- ions were added before subjecting it to a 100,000 ns production run. The RMSD plot obtained for the RBD of wild-type SARS-CoV-2-Lupeol complex (black color) showed a convergence at 20,000 ps with ~0.15 nm difference in the ligand-bound state (Figure 9). Initially, the protein-ligand complex remained intact with the RMSD value between 0.1 and 0.15 nm. After attaining the equilibrium state at 20,000 ps, the RMSD value for the complex continued to be stable between 0.15 and 0.2 nm throughout the simulation period. At the end of the energy minimization, the lowest potential energy conformation was obtained with an energy value of–6.597KJ/mol. Likewise, the RMSD of Lupeol (blue) showed deviations in the range of 0.05 and 0.075 nm, which were minimum and consistent throughout the simulation period, when compared to that of the complex. Similarly, the mobility of the plant compound in the complex with the protein (residue-wise) calculation performed in the trajectory was visualized as an RMSF plot. Upon further analysis, the RMSF of the Lupeol-Wild-type S RBD complex displayed moderate fluctuations in the protein backbone Cα atoms (black color) with an RMSF value of ~0.1 nm, in the ligand-bound state (Figure 10). However, the ligand RMSF plot showed fluctuations for Lupeol (green) in the range of 0 and 0.15 nm, which were relatively minimum when compared to that of the Wild-type S RBD-Lupeol complex. Furthermore, the radius of gyration plot obtained illustrates that the protein in the docked complex showed no conformational changes, maintaining the Rg value between 1.81 nm and 1.87 nm throughout the simulation (Figure 11), thus confirming the higher compactness of ligand.

### 2.6. Delta Spike RBD-Betulin Complex

**Figure 12:**
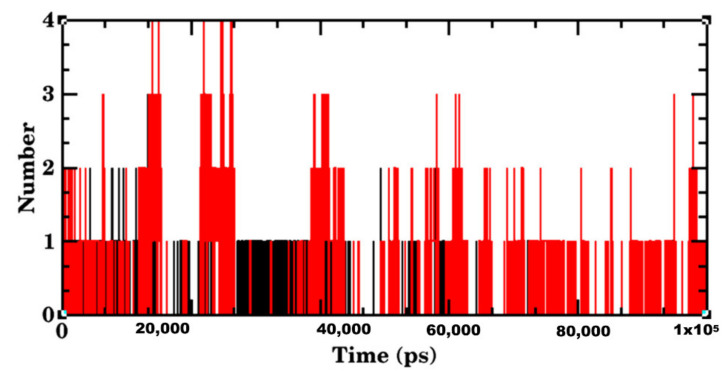
H-bonds plot showing the number of hydrogen bonds that stabilized the protein-ligand complexes of wild-type S RBD-Lupeol (black) and Delta S RBD-Betulin (red).

The equilibrated simulation system of Delta S RBD-Betulin contained 34 atoms with 15,241 water molecules, and 3 Cl- ions. The lowest potential energy of the system obtained from energy minimization was calculated to be −7.5534 × 10^−5^ J. To determine the structural changes occurred in the complex, the trajectories obtained were analyzed. The RMSD plot of the receptor-ligand complex (in red color) showed stable binding orientations. The RMSD value for the complex remained stable between 0.2 and 0.3 nm after attaining the equilibrium state at 20 ns with no further drifts. However, the Delta S RBD-betulin complex exhibited no significant changes in the RMSD value less than 0.2 nm, which indicated the stable state of the protein during the simulation. (Figure 9). Similarly, the RMSD of Betulin (green) was 0.05 nm, which was the lowest and consistent throughout the simulation period, when compared to that of the complex and Lupeol. Unlike the wild-type S RBD-Lupeol complex, the Delta S RBD-betulin complex was stabilized further at 40 ns, after which its stability increased with the RMSD value of 0.1 nm during the rest of the simulation period. Similarly, the RMSF of the complex was generated to analyze the residual atomic fluctuations of the protein’s atoms in the presence of betulin. As shown in Figure 10, minimal fluctuations are indicated by the peaks in red. The average RMSF value for the complex in the ligand-bound state was computed to be 0.1 nm. The lowest RMSF value indicated that the protein after binding to the ligand did not fluctuate more from their mean structure. Nevertheless, the ligand RMSF plot showed fluctuations for Betulin (blue) in the range of 0 and 0.1 nm, which were relatively the lowest when compared to that of the Wild-type S RBD-Lupeol complex and Lupeol. Additionally, the potential energy of the Delta S RBD-Betulin complex remained equal/differed from that of the wild-type, which was calculated as −7.553 KJ/mol. Added to this, the radius of gyration, which illustrates the overall distribution and stability, was comparatively better in the Delta complex than that of the wild-type complex (Figure 11). Further, intermolecular interactions are also considered an important factor in assessing the stability of a protein-ligand complex during simulation. Hence, the hydrogen-bonding pattern in the two complexes was studied using the protein and ligand criteria of gmx_hbond utility of GROMACS. Initially, both the complexes showed more of hydrogen bonds, which were reduced (one H-bond in the wild-type S RBD-Lupeol complex and four in the Delta S RBD-Betulin complex) in the docked complexes (Figure 12) during the simulation run. Thus, MD simulation results correlated with the docking results in terms of hydrogen bond formation, which were observed in the dynamic state.

Further, we performed a MM-PBSA analysis to calculate the thermodynamics parameters of the protein-ligand complexes, such as Van der Waals, electrostatic, polar solvation, SASA, and binding free energies (Table 3). The binding energy of the wild-type and Delta S RBD structures with the Lupeol and Betulin computed were −25.73 kJ/mol and −24.48 kJ/mol, respectively. These data together suggest that Lupeol and Betulin may bind with the S RBD of the two variants efficiently and therefore could be considered for further analysis.

### 2.7. Biological Activity, Drug-Likeness, and Pharmacokinetic Profiles of the Identified Lead Compounds

A PASS prediction of the investigated natural compounds discerned that Lupeol and Betulin possess a moderate biological activity with a Pa score of 0.667 and 0.647, respectively. The Pa score of Hypericin was 0.460 (Table 4). Hence, Lupeol and Betulin among the five compounds are more likely to be experimentally active. Likewise, the drug-like compounds were recognized based on Lipinski’s rule of five using SwissADME. It predicted that Lupeol and Betulin, having a higher bioavailability score, of 0.55, complied with Lipinski’s rules, with only one violation (Xlog P3 value > 5). In contrast, the number of hydrogen bond donors and acceptors formed by Hypericin was high, violating two of Lipinski’s drug-likeness criteria as in Remdesivir and Azithromycin (Molecular weight > 500 Da and H-bond acceptors > 10) (Table 5). Besides, the bioactive compounds were cataloged based on their predicted ADMET properties. Although the gastrointestinal absorption was low, the solubility value of Lupeol and Betulin was in the acceptable range and did not pass through the blood–brain barrier. These pentacyclic triterpenoid compounds showed no hepatotoxicity (Table 5). On the other hand, Hypericin was predicted as hepatotoxic to one of the cytochrome P450 isoforms. Our results cumulatively indicated that Lupeol and Betulin possess significant drug-like properties in terms of the diverse parameters assessed.

## 3. Discussion

The accumulation of single nucleotide mutations in the viral genome has led to the emergence of several SARS-CoV-2 variants (1). Although massive efforts have been taken to vaccinate the world population, the knowledge about the period of protective immunity elicited by the COVID-19 vaccines remains largely vague [14,15]. COVID-19 vaccines are believed to have reduced the risk of disease severity and mortality; however, newly arising SARS-CoV-2 variants may evade vaccine-induced immunity or infect the immunosuppressed patient population. Moreover, the spread of the disease is fueled by the newly emerged variants even among vaccinated individuals [16] and different age groups [17]. Besides this, several existing synthetic antiviral molecules, which have entered clinical trials, have not shown any proven efficacy against SARS-CoV-2 and its variants so far [18]. The unavailability of SARS-CoV-2-specific antiviral drugs emphasizes the need for the identification of new leads. 

Although COVID-19 disease transmission to humans is a resultant of the latest evolutionary event, the number of mutations in the encoded spike protein has been rapidly increasing [19,20]. As a result, a high diversity of mutants in the viral Spike protein are reported by recent epidemiological studies. Among the variants reported, strains like Alpha, Beta, Gamma, and Delta of B.1.1.7, B.1.351, and other similar lineages, respectively, have been associated with an increased stability and infectivity of the virus [13,21]. The Delta variant, which originated in India in December 2020 and later spread to different parts of the world, harbors fifteen mutations: T19R, (V70F*), T95I, G142D, E156del, F157del, R158G, (A222V*), (W258L*), (K417N*), L452R, T478K, D614G, P618R, and D950N in the spike protein. The N501Y mutation, which is located on the RBD, was common to all the SARS-CoV-2 variants except Delta (13). Of the mutations mentioned above, K417N, L452R, and T478K are the substitutions located in the S RBD region of the Delta variant. 

While the disease continues to be transmitted by the latest variants, COVID-19 infections has been treated symptomatically without specific drugs until today. A recent study, which investigated the drug safety in COVID-19 patients who received Remdesivir for their treatment, suggested that the antiviral drug might cause hepatocellular injury [22]. Another randomized, double-blinded, placebo-controlled, and multicenter trial conducted in a large Chinese population for the treatment of COVID-19 has shown that the association between the Remdesivir treatment and its clinical benefits was not statistically significant [23]. For these reasons, discovering novel therapeutic drugs is of paramount importance for inhibiting the rapidly evolving essential proteins of SARS-CoV-2 viruses. In general, targeting the Spike glycoprotein of similar RNA viruses, like Ebola, has shown promising therapeutic outcomes. Thus, the Spike glycoprotein has become a principal target owing to its critical role in the SARS-CoV-2 cell entry mechanism [24]. Besides, Indian medicinal plants have been known for their remarkable anti-inflammatory, anti-allergic, anti-bacterial, anti-fungal, immunomodulatory, and anti-viral inhibitory effects against a wide range of pathogenic infections [25]. Earlier, the extracts and phytochemical compounds of Decalepis hamiltonii, Acacia modesta, Hypericum perforatum, Phylanthus amarus, and Phyllanthus emblica have shown remarkable efficacy against viruses like chikungunya, hepatitis, herpes, and bronchitis [26,27,28,29,30]. Hence, new antiviral strategies must take advantage of the antiviral inhibitory properties of naturally occurring phytochemical compounds instead of focusing only on synthetic substances. The current study used an *in silico* approach to screen a library of fifty plant-based antiviral compounds that are commonly present in Indian medicinal plants and identify potential hits against wild-type and Delta forms of SARS-CoV-2. We investigated the binding affinities of the selected phytochemical antiviral inhibitors, which could describe their binding mode in the active sites of the target protein. We further re-docked the top-ranked natural compounds against the spike RBD of wild-type and Delta SARS-CoV-2 in order to explore their plausible binding affinities.

In the present study, the low RMSD value of the superimposed RBD structures in the two S proteins and between the crystal structured and the modeled Delta S RBD implied that the two conformers shared close structural similarities. However, the amino acid residues comprising the RBD region of the wild-type spike protein were previously annotated to form an α-helix. The residues resulting from the substitutional events in the Delta RBD did not prefer α-helices and β-strands as secondary structural elements. The increase in the number of polar amino acids in the S RBD of the Delta variant might be responsible for its increased stability and ACE2 binding affinity. Our observation was in accordance with previous studies, which reported that the RBD of the Delta variant showed stronger interactions with the ACE2 receptor, unlike any other SARS-CoV-2 variants [31]. These critical mutations, which are associated with a higher binding affinity and stability in the Delta variant, could have possibly increased the viral infectivity and pathogenicity [1]. Thus, the obtained molecular insight vividly describes the impact of the amino acid substitutions that influenced Delta S RBD’s receptor-binding affinity.

Our cumulative analysis of the molecular docking and dynamics simulation results obtained inferred that Lupeol and Betulin are the potential hits. They showed predicted binding interactions in the active site of wild-type and Delta S RBD. Lupeol performed slightly better than Betulin in the case of wild-type S RBD. Lupeol has exhibited a broad spectrum of biological activities such as anti-proliferative, anti-angiogenic, anti-inflammatory, hypocholersterolemic efficacies, including antiviral activity against hepatitis B viral replication, and the herpes simplex virus under in vitro and in vivo conditions, as confirmed by several studies in the past [32,33]. In the case of Delta S RBD, our *in silico* analysis ranked Lupeol as the second-best compound, which interacted with the target at two sites. Our results were in line with the previous experimental findings on the antiviral activities of Lupeol [34,35,36]. Another study, which treated Vero E6-propagated SARS-CoV-2 cells using Lupeol, in combination with a few other natural compounds, discovered that the compound synergistically inhibited the surface protein of the pathogen. Upon the direct or indirect mode of interaction(s) with the virions through its amino acid moieties with glycoprotein fusion [6]. Similarly, Betulin, which showed a significant binding affinity to Delta S RBD, is an active antiviral triterpenoid by suppressing the reproduction of cells infected with herpes simplex virus type I and influenza FPV and Echo 6 viruses in the earlier in vitro experimental studies [37]. Betulin and its derivatives are known to inhibit various enveloped and non-enveloped viruses, like the herpes simplex virus, influenza A virus (H1N1, H7N1, H3N2), influenza B, respiratory syncytial virus, and picornavirus under in vitro and in vivo experimental conditions [38]. All the five phytochemical compounds formed at least one hydrogen bond interaction concordantly with the active site residues, such as ARG403, TYR453, GLN493, SER494, GLY496, GLN498, THR500, ASN501, and TYR505 of the wild-type and Delta S RBD. It is worth mentioning that hydrophobic interactions accounted for more than 50% of the protein-ligand interactions of all the compounds with the S RBD of the wild-type and Delta variants. The amino acid residue LYS of the wild-type RBD, which was substituted with ASN at the 417th position, was predominantly involved in the hydrophobic interactions of all the protein-ligand complexes. Additionally, TYR449, LEU455, TYR495, PHE497, ASN501, and GLY502 repeatedly participated in the hydrophobic interactions that stabilized the protein-ligand complexes. Some of the recent computational and experimental studies have reported the aforementioned amino acids as key catalytic site residues on the Spike RBD for drug development [23,36,37]. The RMSD, the RMSF of the complexes and the ligands, the Rg of the complexes, the H-bond patterns, and the binding free energies obtained for the two complexes together emphasized that the two phytochemical compounds possessed significantly higher binding affinity towards the target S RBD of the wild-type and Delta SARS-CoV-2 variants. 

Previous studies have provided evidence that naturally occurring triterpenoids like Betulin and its derivatives inhibits the binding activity of SARS-CoV-2 Spike RBD with the ACE2 receptor [38]. In addition, recent experimental studies have demonstrated the antiviral effect of Betulin on herpes simplex viruses (HSV) types 1 and 2. Although the mechanism of action of Betulin is not sufficiently studied, it is suggested to be involved in the disruption of the HSV replication cycle [39]. In another experiment conducted to discover the underlying mechanism of the anti-HSV activity of Lupeol and Betulin, the compounds were observed to significantly inhibited the replication mechanisms in the exposed HSV-infected cells, both in acyclovir-sensitive and acyclovir-resistant strains. The study elucidated the direct-virus interactions that were mediated by the antiviral activity of the two pentacyclic triterpenes [40]. Further, a clinical trial experiment conducted with the birch bark extract of Betulin for treating chronic hepatitis C virus (HCV) revealed that the levels of alanine transaminase and HCV mRNA significantly decreased. It was noticed that abdominal discomfort and dyspepsia were significantly reduced in the study participants [41]. Overall, three biological mechanisms that could be considered to account for the key action of effective antiviral natural molecules like Lupeol and Betulin include: (i) viral adsorption, as they might inhibit the attachment of the virus to the infected host-cell membrane, thereby blocking the viral entry, (ii) virucidal activity, as they might be involved in the direct action to destroy or deform the viral surface proteins, and (iii) inhibition of viral replication. The aforementioned mechanisms either independently or in combination could be crucially responsible for their antiviral activities [7]. Molecular dynamics simulation has been a reliable approach to gain meaningful insights into the modifications happening in a receptor’s interaction with its small molecule at a particular time scale. MD simulations running for longer periods are believed to provide precise information about a protein-ligand complex [42]. As inferred by molecular dynamics, Lupeol and Betulin formed stable complexes with the S RBD of both the wild-type and Delta variants. Finally, both compounds possessed favorable ADMET profiles, as shown by the results of Swiss-ADME. Hence, the triterpenoid compounds could be strong drug candidates with a more significant inhibitory effect than the synthetic controls.

## 4. Materials and Methods

### 4.1. Viral Proteins

We retrieved the three-dimensional structure of the wild-type SARS-CoV-2 Spike protein-human ACE-2 receptor complex (PDB ID 6M0J, resolution = 2.45 Å) from the RCSB-Protein Data Bank [43]. After removing all of the substructures and the co-crystallized ACE2 receptor from RBD, the PDB coordinates of the atoms constituting the amino acids were saved in PDB format for further analysis. During the present study, there was no crystal structure available for the Spike protein of the SARS-CoV-2 Delta variant. Hence, the crystal structure of the Spike protein of the Delta variant was computationally modeled by inserting the above-mentioned amino acid substitutions in the RBD structure of 6M0J using Swiss PDB Viewer [44]. The mutant structure was energy-minimized by applying AMBER ff99SB in UCSF-Chimera 1.15 [45].

### 4.2. Superimposition of Spike RBD in SARS-CoV-2 Wild-Type and the Delta Variant

To check the compatibility, structural, and conformational changes in the binding pockets of Spike RBD in the wild-type and Delta forms, their three-dimensional structures were superimposed using the PyMol molecular visualization, and their root mean squared deviation (RMSD) value was calculated [46]. Additionally, the reliability of the modeled Delta S RBD was superimposed with a recently available crystal structure of Delta S RBD that was retrieved from PDB (7W92) using PyMol ‘super’ functionality.

### 4.3. Secondary Structure Prediction of S RBD ofthe Wild-Type and Delta SARS-CoV Strains

The secondary structural elements such as alpha-helix, beta-sheet, loops, and coils of the proteins were predicted. To this aim, the FASTA sequences of wild-type (6M0J) and Delta (mutated 6M0J) S RBD were submitted to the secondary structure prediction tool, PSIPRED [47]. 

### 4.4. Antiviral Phytochemical Compounds

Fifty secondary metabolites with potential antiviral properties were chosen after carrying out a detailed literature survey. The phytochemical compounds selected were of Indian origin, and the list of plants and their bioactive compounds are provided in Table 6. The compounds selected belonged to different phytochemical categories like alkaloids, terpenoids, polyphenols, and flavonoids. The two/three-dimensional conformers of the small molecules in the chosen ligand set were downloaded from the NCBI PubChem-Compound database [47,48,49,50,51,52,53,54] in SDF format, and were subsequently converted to PDB format using the OpenBabel chemical file format converter [55]. 

### 4.5. Virtual Screening of Potential Antiviral Plant Compounds

We employed a virtual screening technique to identify natural compounds that possess inhibitory potential against the spike protein of the SARS-CoV-2 wild-type and Delta variants using the open-access standalone tool, PyRx [68]. To this aim, we began the virtual screening process with the library of previously known fifty phytochemical compounds that were prepared in the previous step. Firstly, the three-dimensional structure of the wild-type SARS-CoV-2 Spike protein was optimized and prepared by adding hydrogen atoms and partial charges, which was subsequently converted to the ‘pdbqt’ format. Secondly, the bioactive compounds were loaded into the PyRx workspace one by one, energy minimized, and converted to the ‘pdbqt’ format for further screening. After feeding the active site residue information, a grid box was set to cover the binding pocket of the target protein with the following dimension in Å: (X, Y, Z) = (70Å, 70 Å, 70 Å) and center (X, Y, Z) = (– 3.528 Å, –30.111 Å, 53.389 Å). Finally, the virtual screening process was launched using the AutoDock Vina tool to recognize the top-ranked compounds based on their binding energy scores. The above steps were repeated for the spike protein of the SARS-CoV-2 Delta variant that was chosen for the study. 

### 4.6. Molecular Docking

We selected a subset of the five best compounds with the lowest binding energy (Lupeol, Betulin, Hypericin, Corilagin, and Geraniin) from the previous step and performed flexible docking using AutoDock 4.2.6 [69]. FDA-approved drugs such as Remdesivir and Azithromycin were used as controls for comparison purposes. Initially, the PDB coordinate file of the wild-type Spike protein was prepared by adding polar hydrogens, partial charges, and atom types and saved in an AutoDock-specific ‘pdbqt’ format. Similarly, ligand optimization was done by cleaning the geometry, minimizing their energies; subsequently, a ligand ‘pdbqt’ file was created. Amino acids such as TYR449, GLN493, SER494, TYR495, GLY496, PHE497, GLN498, ASN501, and TYR505 were specified as active site residues [70]. Docking grid box dimensions were fixed around the amino acid residues at 90 × 90 × 90 Å of the X, Y, and Z coordinates with 0.375 Å spacing, and the grid center at (–3.750 Å, –33.389 Å, 58.250 Å), respectively. All the necessary files, such as the macromolecule and ligand (.pdbqt) files, grid parameter (.gpf) file, and docking parameter (.dock) file, were generated on the AutoDock GUI platform and prepared for downstream analysis. The genetic algorithm (GA) was chosen to evaluate the parameters, while the Lamarckian GA was employed for running docking simulations. AutoDockTool, AutoGrid, and other components of AutoDock tools were used to perform molecular docking and the binding mode analysis of the receptor-ligand interactions, which generated the ten best binding conformations (Supplementary Figure S1). As a selection criterion, the lowest binding energy (in kcal/mol) was used to choose the conformers for further analysis. The same protocol was repeated for the Spike protein of the Delta variant and the chosen ligand set. The obtained protein-ligand complexes were visualized using the PyMol visualization system to get an insight into their binding interactions.

### 4.7. Molecular Dynamics (MD) Simulation in Water

MD simulation was performed to evaluate the structural dynamics of the ligands bound to the active site residues of the target proteins. The docked complexes of SARS-CoV-2 wild-type and Delta S RBD with Lupeol and Betulin were subjected to a 100 ns MD simulation using the GROMACS 2020.1 package [71]. Wild-type S RBD-Lupeol and Delta S RBD-Betulin complex structures were taken as initial conformations for the corresponding simulations. Briefly, protein topology files were built using the pdb2gmx topology builder (built-in feature) of GROMACS. Similarly, the topologies of Lupeol and Betulin were generated with the help of the CHARMM general force field (CGENFF) web server [72] employing the CHARMM force field. The prepared protein and ligand topology files were imported into a unit cell to define a single system. The created complex in the system was subsequently solvated in a predefined water environment using the ‘water TIP3P’ model. The docked complexes of wild-type and mutant proteins were solvated in a dodecahedron box system and subsequently neutralized by adding 2 and 3 Cl- ions, respectively. Energy minimization was performed using a CHARMM36 all-atom type force field and the steepest descent algorithm, with the Fmax value set to no greater than 1000 kJ mol^−1^ nm^−1^ [73]. Subsequently, the energy-minimized system was equilibrated using the NVT and NPT ensembles for 100ps. The temperature was maintained at 310K with constant pressure at 1.01325 bar [74,75]. Finally, 100 ns simulations for each system were performed for further analysis. The dynamic behavior of the entire simulated systems was evaluated by employing root mean square deviation (RMSD) and root mean square fluctuation (RMSF) for both protein backbone Cα atoms and also for ligands. In addition, the radius of gyration (Rg) for the complexes, and the H-bonds for protein and ligand criteria, were analyzed. These tasks were performed using the standard tools implemented in GROMACS. Finally, the generated trajectory plots were visualized and analyzed using Xmgrace [76]. Furthermore, the molecular mechanics Poisson–Boltzmann surface area (MM-PBSA) method was used to re-score the binding free-energies of the wild-type S RBD-Lupeol and Delta S RBD-Betulin complexes using the g_mmpbsa tool of GROMACS [77]. The binding free energy for the two complexes was computed using the following equation:

△G_binding_ = G_complex_ − (G_protein_ + G_ligand_)

In the above equation, G_complex_ indicates the energy of the phytochemical compound bound to the S protein in each of the docked complexes, while G_protein_ and G_ligand_ indicate the energies of protein and ligand in the solvated environment, respectively.

### 4.8. Prediction of Biological Activity for the Screened Natural Compounds

We used the ‘prediction of activity spectra for substances’ (PASS) online server [78] to predict the biological (antiviral) activity of the drug-like compounds based on their structural features. In other words, the prediction strategy of PASS is based on the principle that the structure formulae of the virtual compounds influence their probable biological activities, including the pharmacotherapeutic effects, biochemical mechanism of action, toxicity, adverse effects, interaction with metabolic enzymes and transporters, structure-activity relationship, and gene expression regulation [79].In this study, SMILES notations of the phytochemical compounds under investigation were given as input for estimating their biological activity.

### 4.9. Prediction of Drug-Likeness Parameters and ADMET Properties for the Identified Plant Compounds with Antiviral Inhibitory Potential

The selected bioactive compounds and the FDA-approved control drugs were screened further based on their drug-likeness and pharmacokinetic ADMET properties by implementing SwissADME and ADMETSAR [80,81]. The cumulative results given by both the tools were profiled and compared. The canonical smiles of the compounds were submitted as input for the prediction. Physicochemical attributes such as molecular weight, number of H-bond donors and acceptors, AlogP, water solubility, and the bioavailability score were predicted to evaluate the drug-likeness of the chosen compounds. The subcellular localization descriptor was used to predict the distribution of the therapeutic molecules in the body. Likewise, predictors such as gastrointestinal absorption, blood–brain barrier permeability, Caco-2 permeability, permeability glycoprotein (P-gp) substrate and inhibitor, and renal organic cation transporter were used to assess the pharmacokinetics attributes. In addition, the drug metabolic interactions of the small molecules with cytochrome P450 enzymes such asCYP450 2C9, CYP450 2D6, CYP450 3A4, CYP450 1A2, CYP450 2C9, CYP450 2D6, CYP450 2C19, and CYP450 3A4 were evaluated. Similarly, the toxicity predictors of the bioactive compounds were used to validate the pharmacokinetic behaviors of the selected drug candidates.

## 5. Conclusions

Taken together, the pentacyclic triterpenoids such as Lupeol and Betulin revealed promising binding affinities towards the Spike protein of both the wild-type and Delta SARS-CoV-2 strains. Disrupting the functions of S RBD and its binding with ACE-2 with the help of potential natural small molecules could decrease the probability of the pathogen to escape even in the event of genetic drifts that occur in response to selective pressure during the prophylactic treatment of COVID-19 disease. Therefore, considering the pivotal role of the spike protein in the regulation of viral entry, pathogenesis, and pathogenesis, Lupeol and Betulin could be promising lead scaffolds for developing cost-effective COVID-19 therapeutics against SARS-CoV-2 variants. Nevertheless, further experimental investigations are required to confirm the efficacy, while accounting for the detailed mechanism of action of Lupeol and Betulin in the cure of the disease.

## Figures and Tables

**Table 1:** Molecular docking results of potential compounds against wild-type S protein.

Compound	Binding Energy (kcal/mol)	H-Bond Interactions	Hydrophobic Interactions
Lupeol	−8.54	TYR505(2.1 Å)	ARG403, ASP405, GLU406, LYS417, TYR453, LEU455, GLN493, TYR495, GLY496, PHE497, GLN498, ASN501, GLY502
Betulin	−8.29	ARG403(1.8 Å), GLU406(2.1 Å), GLY496(2.1 Å)	ASP405, LYS417, TYR453, LEU455, GLN493, SER494, TYR495, PHE497, GLN498, ASN501, TYR505
Hypericin	−8.13	GLN493(2.1 Å), GLN493(2.2 Å), GLN493(2.8 Å), SER494(2.0 Å), GLN498(2.3 Å), GLN498(2.6 Å), ASN501(2.1 Å)	TYR449, TYR495, GLY496, PHE497, THR500, TYR505
Corilagin	−7.21	TYR453(2.1 Å), GLN493(2.0 Å), GLN493(2.5 Å), SER494(2.3 Å), ASN501(2.5 Å), GLN498(2.3 Å)	ARG403, GLY446, TYR449, TYR451, TYR495, PHE497, THR500, GLY502, TYR505
Azithromycin	−7.17	ARG403(2.0 Å), ASP405(2.0 Å), ASP405(2.2 Å), ASN501(2.1 Å)	GLU406, ARG408, LYS417, TYR453, LEU455, PHE456, GLN493, GLY496, GLN498, THR500, GLY502, VAL503, GLY504, TYR505
Remdesivir	−6.69	ARG403(2.1 Å), ARG403(2.4 Å), TYR453(2.1 Å), GLN493(1.8 Å), GLN493(2.7 Å), SER494(2.9 Å)	GLU406, GLN409, LYS417, ILE418, TYR495, GLY496, PHE497, GLN498, ASN501, GLY502, TYR505
Geraniin	−6.03	TYR453 (2.8 Å), GLY496(2.6 Å), THR500(2.4 Å), ASN501(1.4 Å), ASN501(2.1 Å), ASN501(2.2 Å), GLY502(2.7 Å)	ARG403, GLN493, SER494, TYR495, PHE497, GLN498, TYR505

**Table 2:** Molecular docking results of potential compounds against Delta S protein.

Compound	Binding Energy (kcal/mol)	H-Bond Interactions	Hydrophobic Interactions
Betulin	−8.83	THR500(1.7 Å), GLN493(2.1 Å), GLN493(2.6 Å), SER494(1.8 Å)	ARG403, TYR453, TYR495, GLY496, PHE497, GLN498, ASN501, GLY502, TYR505, GLN506
Lupeol	−8.68	GLN493(1.9 Å), SER494(1.9 Å)	ARG403, TYR495, GLY496, PHE497, GLN498, THR500, ASN501, GLY502, TYR505, GLN506
Hypericin	−8.59	TYR453(2.8 Å), GLN493(2.3 Å), SER494(2.4 Å), SER494(2.9 Å), GLY502(2.3 Å), TYR505(2.1 Å), TYR505(2. 3 Å)	ARG403, TYR449, TYR495, GLY496, PHE497, GLN498
Corilagin	−7.35	ARG403(1.8 Å), ARG403(2.1 Å), ARG403(2.6 Å), GLU406(2.2 Å), TYR421(2.3 Å), TYR421(4.3 Å)	GLN409, ASN417, ILE418, TYR453, PHE456, ARG457, GLN493, TYR495
Azithromycin	−7.31	GLU484(2.4 Å), PHE490(2.1 Å), GLY496(2.1 Å)	ARG403, GLY446, TYR449, TYR453, LEU455, PHE456, LEU492, GLN493, SER494, TYR495, PHE497, GLN498, THR500, ASN501, GLY502, TYR505
Remdesivir	−6.92	GLN493(2.1 Å), GLN498(2.7 Å), TYR505(2.8 Å)	ILE402, ARG403, TYR449, TYR453, SER494, TYR495, GLY496, PHE497, THR500, ASN501, GLY502, PRO507
Geraniin	−5.91	GLY496(2.2 Å), GLN498(1.6 Å), GLN498(1.9 Å), GLN498(2.2 Å), GLN498(2.6 Å), GLN498(2.8 Å), TYR500(2.1 Å), GLY502(2.6 Å), TYR505(2.2 Å)	ARG403, ASN439, SER443, TYR449, TYR453, PRO499, TYR495, PHE497, ASN501, VAL503, GLN506, PRO507

**Table 3:** Comparison of binding free energies and individual energies of Lupeol and Betulin calculated by MM-PBSA.

Complex	△E_Van der aals_(kJ/mol)	△E_Electrostatic_ (kJ/mol)	△E_polar_ (kJ/mol)	SASA (kJ/mol)	△G_bind_ (kJ/mol)
Delta S RBD-Betulin	−36.13	2.14	13.28	−5.02	−25.73
Wild-type S RBD-Lupeol	−26.91	−5.72	15.44	−7.29	−24.48

**Table 4:** PASS scores predicted for the selected phytochemical antiviral inhibitors.

S. No.	Compound	Pa	Pi
1.	Lupeol	0.667	0.008
2.	Betulin	0.647	0.001
3.	Hypericin	0.460	0.008
4.	Corilagin	0.401	0.015
5.	Geraniin	0.71	0.003

**Table 5:** Drug-likeness and ADMET predicted for the selected antiviral inhibitors using SwissADME and ADMETSAR.

Sl.No.	Descriptor	Lupeol	Betulin	Hypericin	Corilagin	Geraniin	Remdesivir	Azithromycin
**Drug-likeness**								
1	Molecular Weight (<500 Da)	426.73	442.73	504.45	634.46	952.65	602.59	749.00
2	AlogP (<5)	8.02	7.00	5.08	– 0.30	– 1.10	2.31	1.90
3	H-bond Donor (5)	1	2	6	18	14	4	5
4	H-bond Acceptor (<10)	1	2	8	11	27	13	14
5	No of Violations	1	1	2	3	3	2	2
6	Bioavailability Score	0.55	0.55	0.17	0.17	0.17	0.17	0.17
**Absorption**								
7	Water Solubility (Log S)	Poorly soluble	Poorly soluble	Poorly soluble	Soluble	Moderately soluble	Moderately soluble	Poorly soluble
8	HIA	HIA+	HIA+	HIA+	HIA+	HIA+	HIA+	HIA–
9	Caco-2 Permeability	Caco-2+	Caco-2+	Caco-2+	Caco-2–	Caco-2–	Caco-2–	Caco-2–
10	BBB	BBB–	BBB–	BBB–	BBB–	BBB–	BBB–	BBB–
11	PGS	NS	S	S	S	S	S	S
12	Renal Organic Cation Transporter	NI	NI	NI	NI	NI	NI	NI
**Distribution**								
13	Subcellular Localization	Lysosome	Lysosome	Mitochondria	Mitochondria	Mitochondria	Lysosome	Lysosome
**Metabolism**								
14	CYP450 2C9 Substrate	S	S	NS	NS	NS	NS	NS
15	CYP450 2D6 Substrate	S	S	NS	NS	S	NS	NS
16	CYP450 3A4 Substrate	S	S	NS	NS	NS	S	S
17	CYP450 1A2 Inhibitor	NI	NI	NI	NI	NI	NI	NI
18	CYP450 2C9 Inhibitor	NI	NI	NI	NI	NI	NI	NI
19	CYP450 2D6 Inhibitor	NI	NI	NI	NI	NI	NI	NI
20	CYP450 2C19 Inhibitor	NI	NI	I	NI	NI	NI	NI
21	CYP450 3A4 Inhibitor	NI	NI	I	NI	NI	NI	NI
**Toxicity**								
22	Hepatotoxicity	NHT	NHT	T	T	T	T	NT
23	AMES toxicity	NAT	NAT	NAT	NAT	NAT	NAT	NAT
24	Carcinogens	NC	NC	NC	NC	NC	NC	NC

**Table 6:** Top-ranked phytochemical compounds selected from the virtual screening process.

Compound Name	Molecular Formula	Structure	Phytochemical Category	Known Antiviral Effect against	References
Lupeol	C_30_H_50_O	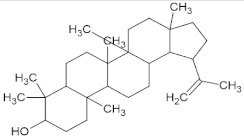	Pentacyclic triterpenoid	Dengue, Herpes, Ranikhet, Encephalomyocarditis, and Semiliki forest viruses	[56,57]
Betulin	C_30_H_50_O_2_	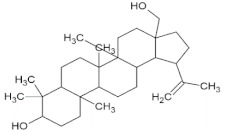	Pentacyclic triterpenoid	Herpes simplex virus type I and HIV type I viruses	[58,59]
Hypericin	C_30_H_16_O_8_	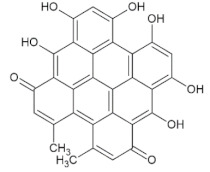	Anthraquinone	HIV type I, Infectious bronchitis virus, and Murine cytomegalovirus	[28,60]
Corilagin	C_27_H_22_O_18_	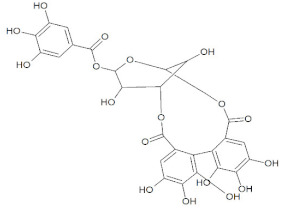	Flavonoid	Hepatitis, Human enterococcus, and Coxsackieviruses	[61,62]
Geraniin	C_41_H_28_O_27_	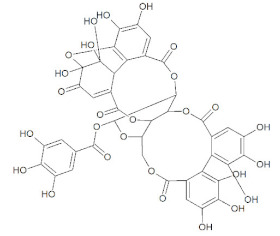	Flavonoid	Influenza A and B, Enterovirus 71 and Dengue virus type 2	[37,63,64,65]
Remdesivir	C_27_H_35_N_6_O_8_P	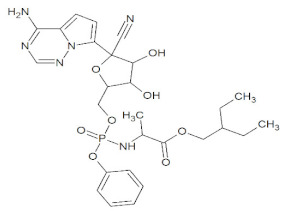	-	Hepatitis C, HIV, Ebola, MERS-CoV, and Respiratory syncytial viruses	[66]
Azithromycin	C_38_H_72_N_2_O_12_	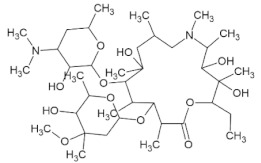	-	Ebola, Zika, influenza H1N1, and rhinoviruses	[67]

## Data Availability

The data presented in this study are available in this article.

## Supplementary Materials

Table S1: Docking based virtual screening results of the 50 phytochemical compound dataset against wild-type Spike protein, predicted by PyRx, Table S2: Docking based virtual screening results of the 50 phytochemical compound dataset against Delta Spike protein, predicted by PyRx, Figure S1: (A) A snapshot of the grid box enclosing the binding pocket of the representative macromolecule (B) Dimensions of the grid box set up around the ligand binding cavity of the protein.

## Sample Availability

Samples of the compounds are not available from the authors.
