# Supplementary material for: Plant-Derived Antiviral Compounds as Potential Entry Inhibitors against Spike Protein of SARS-CoV-2 Wild-Type and Delta Variant: An Integrative in SilicoApproach
Source: Molecules. 2022 Mar 8;27(6):1773. doi: 10.3390/molecules27061773 (PMC8949152; doi:10.3390/molecules27061773)
Supplement: Supplementary file 1 [file molecules-27-01773-s001.zip › molecules-1574752-supplementary.pdf]

Supplementary Material

Table S1 Docking based virtual screening results of the 50 phytochemical compound dataset against wild-type Spike protein, predicted by PyRx.

Sl No.	Compound	Binding energy (kcal/mol)
1	Lupeol	−9.4
2	Betulin	−9
3	Hypericin	−8.8
4	Corilagin	−8.7
5	Rutaecarpine	−7.8
6	Geraniin	−7.8
7	Rutin	−8.5
8	Hyperoside	−8.5
9	Mulberroside C	−8.5
10	Pentagalloylglucose	−8.3
11	Chebulic acid	−8.2
12	Mimusopic acid	−8.1
13	Berberine	−8.0
14	Neoandrographolide	−7.9
15	Chebulaigic acid	−7.9
16	Chebulinic acid	−7.9
17	Quercetin	−7.9

18	Kaemferol	−7.9
19	Germacrone	−7.8
20	Baicelein	−7.8
21	Ellagic acid	−7.7
22	Epigallocatechin gallate	−7.7
23	Glycyrrhizic acid	−7.7
24	Fisetin	−7.7
25	Palmitine	−7.7
26	Torvoside H	−7.7
27	Myricetin	−7.6
28	Rosmarinic acid	−7.5
29	Cordycepin	−7.4
30	Andrographolide	−7.4
31	Carvacrol	−7.3
32	Apigenin	−7.4
33	Piperine	−7.4
34	Curcumin	−7.2
35	Quinic acid	−7.1
36	Thymol	−7.1
37	Eugenol	−7.0

38	P-Coumaric acid	−6.9
39	Gingerol	-6.3
40	Gallic acid	−5.3
41	Ferulic acid	−5.1
42	E-cinnamaldehyde	−5.0
43	Menthol	−4.5
44	Ajoene	−3.7
45	Allicin	−3.4
46	Methyl allyl thiosulfinate	−3.3
47	Diallyl trisulfide	−3.2
48	Gingerone A	−3.0
49	Ursolic acid	−2.9
50	Emblicanin A	−2.9

Table S2 Docking based virtual screening results of the 50 phytochemical compound dataset against Delta Spike protein, predicted by PyRx.

Sl No.	Compound	Binding energy (kcal/mol)
1	Lupeol	−7.5
2	Betulin	−7.5
3	Corilagin	−7.4
4	Hypericin	−7.4
5	Rutaecarpine	−7.4
6	Geraniin	− 7.3
7	Rutin	−7.2
8	Hyperoside	−7.0
9	Pentagalloylglucose	−6.9
10	Mulberroside C	−6.8
11	Mimusopic acid	−6.8
12	Chebulic acid	−6.8
13	Kaemferol	−6.7
14	Quercetine	−6.7
15	Neoandrographolide	−6.3
16	Berberine	−6.2
17	Germacrone	−5.8
18	Ellagic acid	−5.6

19	Glycyrrhizic acid	−5.5
20	Fisetin	−4.9
21	Palmitine	−4.8
22	Torvoside H	−4.8
23	Myricetin	−4.4
24	Chebulagic acid	−4.1
25	Chebulinic acid	−3.6
26	Rosmarinic acid	−3.6
27	Baicalein	−3.6
28	Cordycepin	−3. 4
29	Epigallocatechin gallate	−3.2
30	Carvacrol	−3.2
31	Piperine	−3.1
32	Curcumin	−3.0
33	Apigenin	−2.9
34	Andrographolide	−2.9
35	Thymol	−2.7
36	Eugenol	−2.6
37	Quinic acid	−2.4
38	P-coumaric acid	−2.4
39	Ferulic acid	−2.3

40	Gingerol	−2.1
41	Gallic acid	−2.0
42	E-cinnamaldehyde	−1.9
43	Menthol	−1.8
44	Ajoene	−1.7
45	Allicin	−1.7
46	Methyl allyl thiosulfinate	−1.7
47	Diallyl trisulfide	−1.6
48	Ursolic acid	−1.6
49	Gingerone A	−1.5
50	Emblicanin A	−1.3

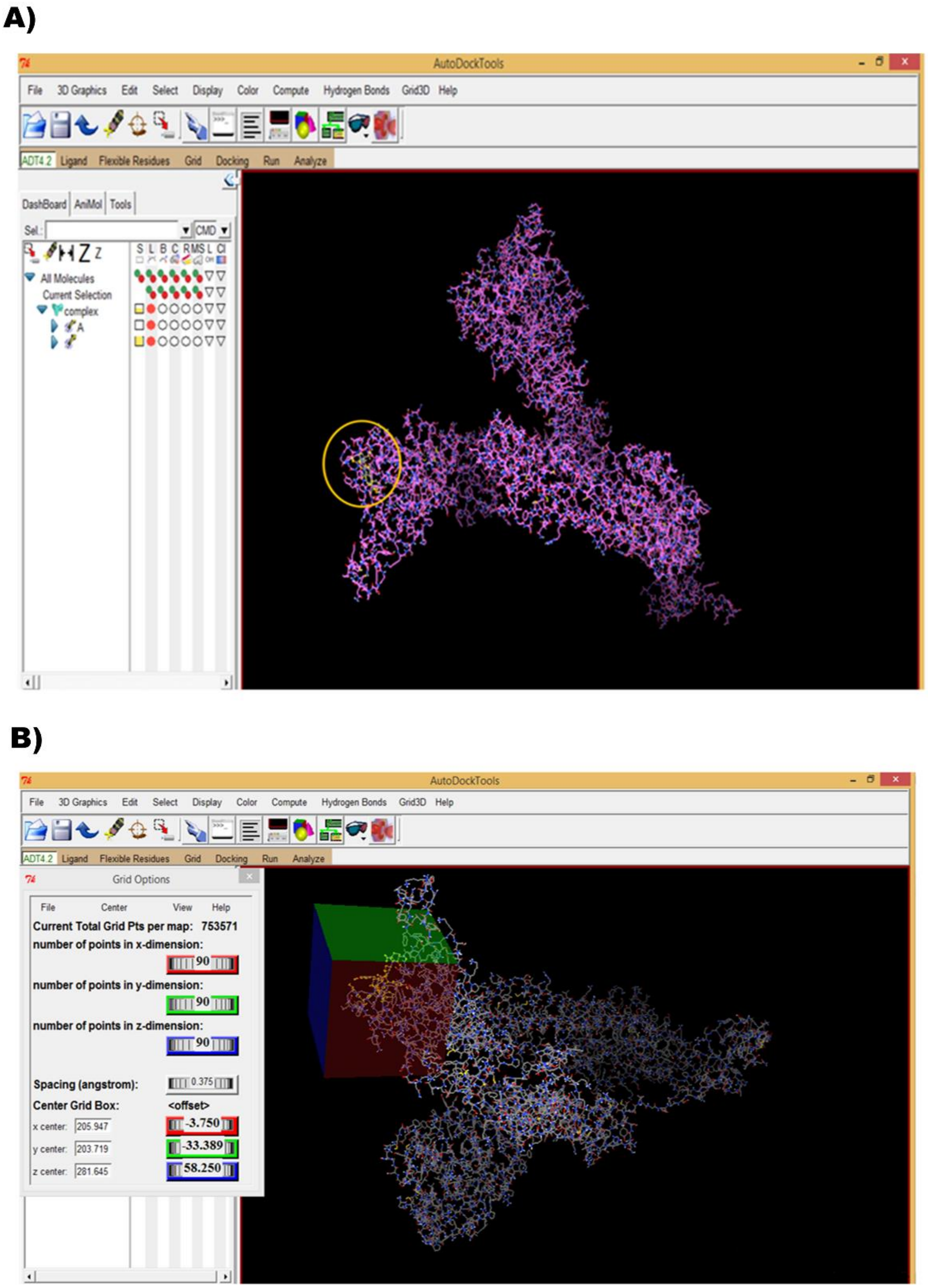

Figure S1. (**A**) A snapshot of the grid box enclosing the binding pocket of the representative macromolecule (**B**) Dimensions of the grid box set up around the ligand binding cavity of the protein.
